# Supplementary material for: Does Cranial Base Angle Make a Difference in the Effectiveness of Functional Orthopedic Treatment? A Retrospective Cohort Study
Source: J Clin Med. 2024 Dec 27;14(1):96. doi: 10.3390/jcm14010096 (PMC11721644; doi:10.3390/jcm14010096)
Supplement: Supplementary file 1 [file jcm-14-00096-s001.zip › jcm-3363447-supplementary.pdf]

Supplementary Table S1. Reference Cephalometric Landmark Explanations.

Sella (S)	Center of the pituitary fossa of the sphenoid bone
Nasion (N)	Most anterior point on the frontonasal suture in the midsagittal plane
Porion (Po)	Most superior point of the external auditory meatus
Orbitale (Or)	Most inferior point on the infraorbital rim
Basion (Ba)	The most posteroinferior point on the clivus
Articulare (Ar)	Intersection of the inferior contour of the posterior cranial base and posterior contour of the ramus
Menton (Me)	The most inferior point of the outline of the symphysis in the midsagittal plane
Posterior nasal spine (PNS)	Posterior spine of the palatine bone constituting the hard palate
Anterior nasal spine (ANS)	Anterior tip of the sharp bony process of the maxilla at the lower margin of the anterior nasal opening
Point A (A)	Deepest point of the curve of the anterior border of the maxilla
Point B (B)	Most posterior point in the concavity along the anterior border of the symphysis
Pogonion (Pog)	Most anterior point on the midsagittal symphysis
Menton (Me)	Most inferior point of the symphysis
Gonion (Go)	Point along the angle of the mandible, midway between the lower border of the mandible and posterior ascending ramus
Gnathion (Gn)	Most anterior inferior point of the contour of the bony menton
Upper incisor (U1)	Tip of the crown of the most anterior maxillary central incisor
Lower incisor (L1)	Tip of the crown of the most anterior mandibular central incisor
Condylion (Co)	Most superior and posterior point of the mandibular condyle

Supplementary Table S2. Cephalometric Measurement Explanations.

Cranial Base Measurements	
N-S-Ba	The angle formed between points N, S, and Ba
S-Ba	Distance between S and Ba point
N-S-Ar	The angle formed between points N, S, and Ar
Ar-N	Distance between Ar and N point
S-Ar	Distance between S and Ar point
S-N	Distance between S and N point
Ar-Go-Me	The angle formed between points Ar, Go, and Me
Ar-Go-N	The angle formed between points Ar, Go, and N
Sagittal and Vertical Skeletal Measurements	
N-Go-Me	The angle formed between points N, Go, and Me
Go-Me	Distance between Go and Me point
SNA	The angle formed between points S, N, and A
SNB	The angle formed between points S, N, and B
ANB	The angle formed between points A, N, and B
Wits	Distance between the projections of point A and B onto occlusal plane
ANS-Me	Distance between ANS and Me point
SN/GoGn	Angle formed between SN and GoGn lines
SN/PP	Angle formed between SN and PP lines
PP/GoGn	Angle formed between PP and GoGn lines
N-Me	Distance between N and Me point
S-Go	Distance between S and Go point
S-Go/N-Me	Angle formed between S-Go and N-Me lines
N-ANS	Distance between N and ANS point
ANS-Gn	Distance between ANS and Gn point
N-Gn	Distance between N and Gn point

CoA	Distance between Co and A point
CoGn	Distance between Co and Gn point
Mx/Md Diff	Maxillary/mandibular difference
Incisor Measurements	
U1/SN	Angle of the long axis of the maxillary incisor with the Sella–Nasion plane
U1/PP	Angle of the long axis of the maxillary incisor with the Palatal plane
U1-NA	Distance between long axis of the maxillary incisor and Nasion-A plane
U1/NA	Angle of the long axis of the maxillary incisor with the Nasion-A plane
L1-APog	Distance between long axis of the mandibulary incisor and A-Pogonion plane
IMPA	Angle formed between Go–Me and the mandibular incisor axis
L1-NB	Distance between long axis of the mandibulary incisor and Nasion-B plane
L1/NB	Angle of the long axis of the mandibulary incisor with the Nasion-B plane
Interincisal Angle	Angle formed by the intersection of the mandibular incisor axis with the maxillary incisoraxis
Overbite	Vertical distance between the tips of the maxillary and mandibular central incisors
Overjet	Horizontal distance between the tips of the maxillary and mandibular central incisors
